# Systems Approaches to Cell Culture-Derived Extracellular Vesicles for Acute Kidney Injury Therapy: Prospects and Challenges

**DOI:** 10.1093/function/zqae012

**Published:** 2024-03-28

**Authors:** David J Lundy, Barbara Szomolay, Chia-Te Liao

**Affiliations:** Graduate Institute of Biomedical Materials & Tissue Engineering, Taipei Medical University, Taipei 235603, Taiwan; International PhD Program in Biomedical Engineering, Taipei Medical University, Taipei 235603, Taiwan; Center for Cell Therapy, Taipei Medical University Hospital, Taipei 110301, Taiwan; Systems Immunity Research Institute, Cardiff University School of Medicine, Cardiff CF14 4XN, UK; Division of Infection and Immunity, Cardiff University School of Medicine, Cardiff CF14 4XN, UK; Division of Nephrology, Department of Internal Medicine, Shuang Ho Hospital, Taipei Medical University, New Taipei City 23561, Taiwan; Division of Nephrology, Department of Internal Medicine, School of Medicine, College of Medicine, Taipei Medical University, Taipei 110, Taiwan; Research Center of Urology and Kidney, Taipei Medical University, Taipei 110, Taiwan

**Keywords:** extracellular vesicle, acute kidney injury, systems biology

## Abstract

Acute kidney injury (AKI) is a heterogeneous syndrome, comprising diverse etiologies of kidney insults that result in high mortality and morbidity if not well managed. Although great efforts have been made to investigate underlying pathogenic mechanisms of AKI, there are limited therapeutic strategies available. Extracellular vesicles (EV) are membrane-bound vesicles secreted by various cell types, which can serve as cell-free therapy through transfer of bioactive molecules. In this review, we first overview the AKI syndrome and EV biology, with a particular focus on the technical aspects and therapeutic application of cell culture-derived EVs. Second, we illustrate how multi-omic approaches to EV miRNA, protein, and genomic cargo analysis can yield new insights into their mechanisms of action and address unresolved questions in the field. We then summarize major experimental evidence regarding the therapeutic potential of EVs in AKI, which we subdivide into stem cell and non-stem cell-derived EVs. Finally, we highlight the challenges and opportunities related to the clinical translation of animal studies into human patients.

## Graphical Abstract

**Graphical Abstract:**
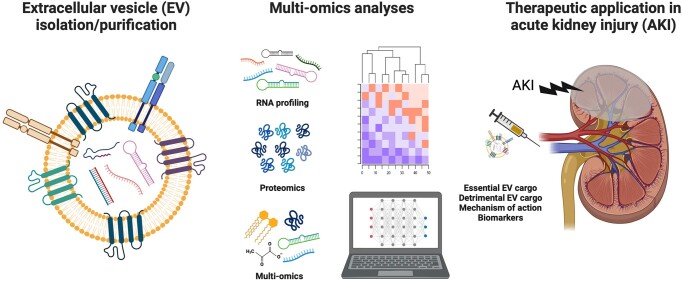

## List of Abbreviations

AEC: amniotic endothelial cell
AI: artificial intelligence
AKI: acute kidney injury
ARF: acute renal failure
BM-MSC: bone marrow mesenchymal stromal/stem cell
BUN: blood urea nitrogen
CKD: chronic kidney disease
CRE: creatinine
ESRD: end stage renal disease
EV: extracellular vesicle
HDF: human dermal fibroblast
I/R: ischaemia/reperfusion
IV: intravenous
ML: machine learning
MSC: mesenchymal stromal/stem cell
NTA: nanoparticle tracking analysis
ROS: reactive oxygen species
SEC: size-exclusion chromatography
TEC: tubular epithelial cell
UC: ultracentrifugation

## Introduction

### Acute Kidney Injury

#### Definitions, Origins, and Treatment of AKI

By clinical definition, acute kidney injury (AKI) is a syndrome of kidney damage resulting in a rapid decline in renal function, a decrease in urine amount, or both.^1^ It is widely recognized that AKI is associated with an increased risk of morbidity and mortality.^2^ The prevalence or incidence of AKI is highly variable in the literature owing to the enrolment from different clinical settings, ie community-acquired AKI versus hospital-acquired AKI.^3^ The exact etiology of AKI is another critical factor, which would considerably influence the outcome data derived from epidemiological studies in this heterogeneous syndrome. In the past, the term “acute renal failure (ARF)” was categorized into 3 major types: pre-renal ARF, intrinsic ARF, and post-renal ARF.^4^ However, this anatomy-based trichotomy is too simplistic and might not reflect the complex nature of ARF. The underlying causes of AKI are diverse and often overlapping. Major pathological conditions leading to AKI syndrome include sepsis, shock, dehydration, post-transplant ischaemic hypoperfusion, reperfusion injury, acute decompensated heart failure, acute liver failure, post-major surgery, nephrotoxins, or drugs (ie non-steroidal anti-inflammatory drugs, contrast agents, aminoglycosides, cisplatin, and aristolochic acid), obstruction of the urinary system (ie ureter stones and strictures), and immune-mediated acute glomerulonephritis.^5–10^ Nowadays, nephrologists and critical care medicine experts use more specific terminology based on specific AKI syndromes, including cardiorenal syndrome, hepatorenal syndrome, sepsis-associated AKI, contrast-associated AKI, etc.^5,6,11,12^ The etiology-based approach has led to deeper understanding of AKI diagnosis, pathogenesis, and treatment, enables more efficient bench-to-bedside communication, and enhances translational research. Complexity and heterogeneity in the origin, pathophysiology, and clinical course of AKI remain major hurdles in developing new treatments. Unfortunately, there are also many limitations of animal experimental studies of AKI, which typically rely on ischaemia-reperfusion (I/R)-based injury or administration of high-dose nephrotoxic drugs, usually in otherwise healthy young rodents. As such, these rarely recapitulate the full clinical scenario of patients with multiple comorbidities.

Globally, the incidence and prevalence of end-stage kidney disease (ESKD) have grown over the past few decades, and AKI is recognized as a major contributing factor for development of chronic kidney disease (CKD).^13,14^ Many AKI patients do not have complete renal recovery during post-AKI follow-up, especially those with severe forms of AKI. Epidemiological observations, clinical studies, and animal studies all show the continuation of pathologic processes, or maladaptive tissue repair, following an episode of AKI, whatever the etiology. Together, these data highlight the complex post-AKI trajectory and the evidence of AKI-to-CKD transition.^15,16^ The underlying mechanisms determining the fate of AKI (injury recovery or progression to chronic fibrosis) remain largely unknown, hindering the development of effective therapeutic strategies. Nevertheless, minimizing short-term and long-term damage at the cellular level, and preserving kidney function following AKI is desirable. Several promising treatment strategies for AKI have been proposed and evaluated in both laboratory and clinical studies. These include small molecules targeting specific biological pathways (ie activation of NLRP3 inflammasome and pyroptosis, endoplasmic reticulum stress response), specific epigenetic regulation (ie miRNAs, long noncoding RNAs, DNA methylation, and histone modification), and others.^17–22^ Cell therapy (including mesenchymal stem/stromal cells (MSCs) and immune cells) or cell-derived products (including cytokines, growth factors, and conditioned medium) have also seen a rapid growth in the past few years.^22,23^

#### Aim of This Review

Here, we present a critical review of the use of cell culture-derived EVs in AKI therapy. We aim to inform and update readers, but also highlight the many unanswered questions that remain in the field, some of which are fundamental to our understanding of EV biology, cargo, mechanisms of action, and overall therapeutic value. To support this, we first introduce basic EV biology and important experimental variables that influence their cargo and activity. We then discuss EV cargo from a systems biology perspective, focusing on miRNAs and proteins, since they have attracted the most research attention. Here, there are multiple hypotheses explaining EV activities, some of which are difficult to reconcile, and we propose that multi-omic studies may provide useful insights. While doing so, we highlight some of the key studies demonstrating the therapeutic use of EVs in AKI therapy. Lastly, we discuss realistic considerations for clinical translation of EVs, including optimizing dose, timing, and delivery routes, and harnessing the knowledge gained from studies of EV cargo and function.

## Extracellular Vesicles (EVs)

### EV Sources for Human Therapeutic Usage

The International Society of Extracellular Vesicles (ISEV) defines EVs as non-replicating, phospholipid bilayer membrane-bound vesicles that are secreted by cells. In this review, we follow MISEV nomenclature recommendations and use “EVs” as an all-inclusive term referring to vesicles of all sizes and cellular origins, though many of the papers we cite used terms such as exosome, microvesicle, or ectosome, which are no longer recommended.^24^ EVs can be isolated from bodily fluids (blood plasma/serum, urine, milk, cerebrospinal fluid, etc.), and they are released from *in vitr o* cultured cells. Blood is particularly rich in EVs and is easily obtained; thus, plasma, serum, and platelet-derived EVs have been widely investigated as biomarkers and as therapeutics in AKI and other diseases.^25–28^ However, it is known that EV yield, cargo, and subsequent biological activities vary depending on donor properties, which can be a limitation of blood-derived EVs.^29,30^ For example, EVs derived from hearts post-myocardial infarction contain pro-inflammatory cargo, which exacerbated injury in recipient mice, and donor age is also reflected in EV cargo and function.^31–33^ On the other hand, cells in culture can be characterized, validated, and grown under defined, xeno-free conditions for EV generation, thus allowing for greater consistency.^34,35^ Furthermore, scientific and legislative frameworks for cell therapy are well established and multiple human clinical trials of MSC therapies have already been completed in AKI and other diseases. As such, this review article focuses mostly on EVs derived from the culture of human MSCs, as we believe these have the highest likelihood of translation to clinical use.

Cell source and culture conditions are of great importance for EV preparations. For example, EVs derived from bone marrow MSCs (BM-MSCs) have less pro-angiogenic potential as their passage number increased.^36^ The same study also demonstrated that seemingly trivial factors such as medium collection frequency can impact the EV yield, with more frequent collections stimulating greater EV production. In addition, culture medium composition, oxygen levels, culture surface properties, and growth environment can all impact EV production and cargo.^37^ For example, EVs derived from MSCs cultured under hypoxia were more protective of ischaemic injury in mice, due to increased amounts of hypoxia-protective cargo.^38^ Such differences in cultural conditions may explain some of the variations seen in results of EV studies, which will be discussed later. Since two-dimensional cell culture on plasticware does not accurately reflect the *in vivo* microenvironment, alternative systems for EV production have also been investigated. For example, cell culture using spheroids or bioreactors has been shown to alter EV yield and cargo.^39,40^ Similarly, we have shown that 3D culture inside a porous scaffold increased EV yield per cell compared to 2D cultures.^41^ Due to the effects of such variables, recommendations for reporting experimental parameters have been released by the ISEV, which can be consulted for more information.^35^

### EV Isolation Methods for Therapeutic Applications

Several isolation methods are commonly used for EV research, including precipitation, filtration, chromatography, affinity-based purification, or centrifugation.^42^ The isolation method must be carefully considered since this impacts the yield, purity, EV integrity, and the subfraction of EVs that are isolated.^43,44^ To purify EVs from conditioned cell culture medium, ultracentrifugation (UC) is the most common choice since it allows processing of large volumes to produce concentrated EVs.^45^ EVs are often co-isolated with other proteins, such as apolipoproteins or albumin; thus, UC-based methods can be refined by combining with affinity-based separation techniques or size exclusion chromatography (SEC) to further improve EV purity.^44,46,47^ However, questions still remain regarding the precise nature of some co-isolated proteins, which may be contaminants based on methodological limitations, or may reflect true biological associations, such as part of the biomolecular corona.^48^

### EV Characterization

EVs must be characterized to validate the isolation and establish the “dose” for administration. This is typically accomplished by measuring the particle count using nanoparticle tracking analysis (NTA), or by quantifying the protein concentration. Ideally, both metrics should be used, since NTA may overestimate EV yield by measuring non-EV particles, and total protein concentrations also measure non-vesicular contaminant proteins, which may have their own biological activities.^44,45^ Since each EV has a finite amount of protein, the particle-to-protein ratio can be used as a metric of purity, with a low ratio indicating the presence of free, non-EV proteins.^49^ Membrane tetraspanins such as CD9, CD81, and CD63 tend to be enriched on EVs, but can also be present as soluble proteins; therefore, EV biogenesis/cargo proteins such as ALIX, HSC70, or ANXA can be specifically assayed to further increase confidence of successful EV isolation.^50^ Syntenin-1 has recently emerged as a suitable biomarker of small EVs from a wide variety of cell sources.^51^ Lastly, electron microscopy can be used to confirm EV morphology. CryoEM is the gold standard since it allows visualization of the phospholipid bilayer forming spherical vesicles, thus distinguishing them from other nanoscale particles such as lipoproteins.^52^ Taking together a combination of particle size, presence of protein markers, and identification of a bilayer membrane, EV isolation can be confirmed. Selecting appropriate isolation and characterization techniques is essential for producing EVs, which could be utilized as a therapeutic product where product identity, purity, safety, and biologic activity should be known and standardized.^53^

### EV Mechanisms of Action in AKI

Since EVs have rich and complex cargo consisting of hundreds of different bioactive miRNAs, proteins, and lipids, they are able to act simultaneously on multiple pathways in different target cells, which are impacted by AKI. For example, during ischaemia/reperfusion (I/R)-induced AKI, there is hypoxic and metabolic/mitochondrial injury, plus additional necrosis and apoptosis of TECs due to trapping of erythrocytes and subsequent toxicity.^54^ Major mechanisms of EV action relevant to AKI therapy include acute protection of parenchymal cells by reducing apoptosis, stimulation of cell proliferation, modulation of inflammation and immune cell recruitment, promotion of endothelial cell angiogenesis, and modulation of matrix remodeling and fibrosis by fibroblasts.^55^ EV cargo constituents are discussed in detail in Section 3, and studies describing their therapeutic activities are discussed in Section 5.

### Comparison of EV-based AKI Therapies to Cell Therapy and Nanocarrier Drug Delivery Systems

Since much of the benefit of cell therapy is derived by paracrine secretions, EVs are an attractive way to harness these effects since they may provide similar therapeutic benefits with less risk than administering live donor cells.^56^ As such, EVs present many advantages compared to cell therapy, both in practical and biological aspects. In practical terms, EV isolation, characterization, and standardization remain overall less burdensome than administration of live donor cells. Efficacy of cell therapy is typically limited by poor delivery, retention, and survival of donor cells at target sites.^57^ This may be due to preparatory cell handling steps (trypsinization, washing, centrifugation, resuspension, etc.), cell death by anoikis following injection, or stresses induced by the hostile post-injury microenvironment.^58^ On the other hand, EVs can be stored frozen for extended periods of time with minimal loss of bioactivity, and they are stable at 37°C.^44,59^ Additionally, since EVs are anuclear, there are no concerns over loss of viability following administration, and there is a lower likelihood of promoting tumorigenesis or mutagenesis.^60,61^ In terms of delivery to target sites, EVs may again be advantageous. MSC biodistribution studies in animals have demonstrated that cells become trapped in small vasculature in the lung and kidney following systemic administration.^62,63^ Additionally, the kidney is a dense tissue with limited space for retention of donor cells, and it has poor cell retention even following direct injection.^57^ On the other hand, the small size of EVs alters their biodistribution and allows passage through delicate structures of the kidney.^64,65^ Some studies have directly compared therapeutic cells against their secreted EVs in an AKI setting. Ren and colleagues evaluated therapeutic effects of amniotic epithelial cells (AECs) and AEC-derived EVs in a mouse I/R kidney model. A number of 1 × 10^6^ cells were administered by intravenous (IV) injection and, unsurprisingly, showed very low integration with the mouse kidney; the majority of AECs were detected in the lung, likely due to entrapment and plugging of microvessels rather than active targeting or specific uptake. However, therapeutic benefits were still observed, presumably due to cell paracrine secretions. AEC-EVs (3 × 10^8^ particles), isolated by ultracentrifugation, recapitulated the same therapeutic benefits as live AECs, including increased animal survival, lowered serum creatinine, reduced kidney cell apoptosis, and improved angiogenesis.^66^ Another important piece of evidence was published by Zhao and colleagues, who compared MSC cell therapy against isolated MSC-EVs using a porcine renal artery stenosis and diet-induced metabolic syndrome-induced AKI model.^67^ EVs were isolated by UC and characterized for size (100-200 nm), protein expression (CD9, CD29, and CD81), and morphology (TEM). Pigs then received MSCs (10^7^) or MSC-EVs (10^11^ EVs) by intra-arterial injection. The EV dose was based on a calculation of the number of EV released from 10^7^ donor cells. Interestingly, both MSCs and MSC-EV treatments showed therapeutic efficacy, but they appeared to act through different mechanisms. MSCs had superior pro-angiogenic effects, while MSC-EVs had stronger anti-apoptotic effects. This may reflect the differences between the cargo of purified MSC-EVs and the overall MSC secretome, the latter of which includes freely secreted growth factors and cytokines. Despite these differences, the overall AKI outcome did not differ between the EV or cell therapy groups, again demonstrating the feasibility of EV-based therapy of AKI.

These 2 studies demonstrate apparent equivalence between EVs and whole cells and illustrate why they are seen as an attractive therapeutic for AKI and other indications.

Due to their sub-micron size, EVs are intuitively comparable to other nanocarrier drug delivery vehicles, such as nanoparticles or liposomes. One apparent advantage of EVs is their superior protection of nucleic acids from degradation and improved intracellular delivery efficiency.^68,69^ For example, a study by Murphy and colleagues showed that EVs were able to deliver RNA therapeutics into cells at an efficiency >10 times higher than lipid nanoparticles.^70^ In the kidney, a study by Reshke and colleagues showed that EVs could carry siRNA to the glomerulus and silence target gene expression more efficiently than the same dose of lipid nanoparticles.^71^ Uptake and passage of nanoparticles by the kidney are highly complex and have been recently reviewed elsewhere.^72^ In addition to more efficient nucleic acid delivery, there is some evidence that EVs may be superior to lipid nanoparticles in terms of intracellular protein delivery, although loading or engineering desired proteins into EVs remains a significant challenge.^73^ Since EV membranes include proteins derived from their originating cell, this appears to allow for a degree of targeting and specific uptake; however, the commonly stated notion of EVs easily crossing biological barriers, such as the blood-brain barrier, has recently been challenged and may have been overstated in the past due to methodological limitations of EV labeling and detection.^65^ Detailed comparisons between liposome and EV biodistributions have also not demonstrated any clear advantages of EVs in terms of their circulation time or organ targeting.^74,75^ Taken together, EVs clearly have some similarities and some advantages compared to cell therapy and nanocarrier therapies. However, their biological origin, heterogeneity in cargo, and unclear links of cargo to function make it challenging to standardize a consistent clinical product in the same way that liposomes or nanoparticles can be mass-manufactured with high consistency.^53^ Additionally, most nanocarriers are dosed using the concentration of the active ingredient, which is difficult to establish for EVs. This will be further discussed in Section 2.7 and Section 3.

### EV Delivery and Uptake by AKI Kidneys

To manifest their activity, the classically described mechanism is that EVs must reach the target site, interact with the desired cell membrane, then deliver their cargo in sufficient concentrations to alter the trajectory of the cell response to injury. The precise mechanisms of EV uptake and cargo delivery are complex, and have been reviewed in detail elsewhere.^76^ In brief, there is evidence that EVs can fuse with target cell membranes, delivering cargo directly into the cytoplasm, or they can bind cell surface ligands and be endocytosed, at which point they may fuse with the endosome membrane and deliver cargo into the cytoplasm, or be subjected to degradation or recycling. Cells such as macrophages, key players in response to AKI, are also capable of engulfing/phagocytosing EVs.^76^ Lastly, there is evidence that EVs can stimulate intracellular pathways by binding cell surface proteins, without the need for internalization.^77^ Thus, assessing “delivery” is a complex issue, and most studies take a whole organ approach and do not specifically measure extracellular/intracellular compartments, cell-specific uptake, or organelle-specific uptake.

**Figure 1.**
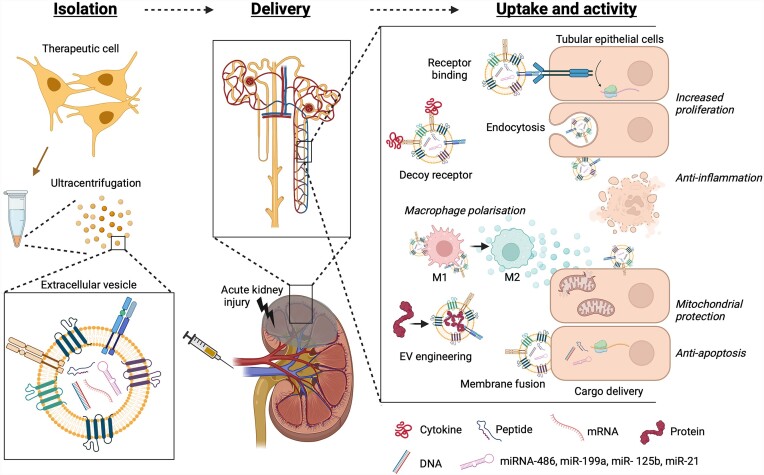
Schematic diagram showing the isolation of therapeutic cell-derived EVs and their cargo, including miRNA, peptides, mRNA, and DNA. Administration to the kidney, EV uptake, and activity are indicated. Multiple mechanisms of EV activity on kidney tubular epithelial cells are shown, including endocytosis and membrane fusion for EV cargo delivery, surface receptor binding to activate intracellular pathways, and the ability to function as a decoy receptor. Indirect activity via macrophage polarization is shown, as is the concept of EV engineering by enriching therapeutic proteins. Lastly, reported mechanisms of therapeutic action are indicated in italics. The figure was made using Biorender.com.

These principles are illustrated in Figure 1, which summarizes EV isolation, cargo, uptake, and mechanisms of action in an AKI setting.

EV uptake after AKI was investigated by Grange and colleagues.^78^ AKI was induced by intramuscular glycerol injection, and MSC-EVs, isolated by ultracentrifugation, were administered at a dose of 200 µg per mouse by IV injection, 3 days post-AKI. The results showed that EVs were detected in the AKI kidney within 10 min after injection and were retained until at least 24 h. Healthy kidneys showed negligible uptake, in agreement with other studies.^65^ This demonstrates that AKI produced an enhanced permeability and retention effect, though it should be noted that EV delivery to the AKI kidney was still an order of magnitude lower than uptake by the liver, lung, and spleen. In terms of cell-specific delivery, proximal TECs appear to uptake EVs following AKI, and they are the most evaluated cell type in *in vitro* or *in vivo* AKI models.^22,79,80^ Interestingly, evidence also indicates that systemically injected EVs can enter the urine without being taken up by kidney cells.^81^ Since the glomerular filtration barrier of healthy kidneys (∼5 nm) is much smaller than EVs (typically >60 nm), this would indicate that EVs cannot normally pass into urine by passive diffusion. However, under certain circumstances of AKI, it is assumed that glomerular pathologies such as endothelial cell damage, podocytopathy, or basement membrane rupture could result in a possible leak of circulating EV into the urinary tract. Unfortunately, there are insufficient studies in this area, and there are no clear conclusions in terms of EV accumulation in urine.^65,81^

### Dosing of EVs for Therapeutic Use in AKI

The dose of administered EVs in AKI research papers is usually based on the total protein, or particle number, which is typically scaled to the body weight of the recipient animal. As mentioned above, both metrics are affected by the purity of the EV sample, and it is now recommended that both protein- and particle-based doses should be reported.^24^ A recent meta-analysis of EV biodistribution studies in rodents highlighted that doses used across different research papers varied by several orders of magnitude, from microgram to milligram doses based on protein, or between 10^8^ and 10^12^ particles.^81^ These concentrations often far exceed physiological levels and are typically given as a bolus dose, making it unclear whether EV targeting is truly being measured, or there is simply accumulation of EVs in organs of clearance, including the liver, spleen, and kidney.^81^ In addition, the active therapeutic component of the EV (key miRNAs, peptides, or EV surface proteins) may be unknown or not quantified, thus the delivered dose of the active ingredient is rarely measured or reported; this will be discussed in the next section. Most research papers use intravenous (IV) injection to deliver EVs to AKI kidneys, and it would be reasonable to assume that more direct routes such as intra-renal artery or direct injection could use lower doses of EVs to achieve the same effects. Administration routes for therapy of AKI are discussed in more detail in Section 6, and the variation of doses is shown in Table 1.

**Table 1.** miRNA bioinformatics tools for prediction and analysis of functional miRNAs and their targetomes.

Desired function	Example resources
miRNA discovery	miRBase, PMRD, EpimiR, AvirmiR, VIRmiRNA, MirGeneDB, miRviewer, miRbase Tracker, mirPub, YM500v2, CoGemiR, mESAdb, miRNEST, Vir-Mir db, and miROrtho
miRNA differential expression	bloodmiRs, mirEX 2.0, PmiRExAt, ExcellmiRDB, miRandola, miREnvironment, and HMED
miRNA deep sequencing tools	miRDeep2 and miRNAkey
miRNA target prediction tools	TarBase, miRTarBase, miRGate, VIRmirTar, MtiBase, miRdSNP, MirSNP, PNRD, PolymiRTS Database, TargetScanS, VIRmiRNA, CSmiRTar, miRecords, miRNA-Target Gene Prediction at EMBL, miRSel, miRSystem, miRWalk, targetHub, miRPathDB, multiMiR, DIANAmicroT Web server v5.0, HOCTARdb, ViTa, miRTar, DIANAmicroT-CDS, MicroCosm Targets, microPIR2, miRDB, and ViTa
miRNA disease association tools	dbDEMC, miRCancer, EpimiRBase, miRStress, DIANA miRPath v.2.0, HMDD, OncomiRDB, and miR2Disease
All-in-one resources	MicroRNA.org, miRNAMap, MtiBase, PMTED, SomamiR DB 2.0, miRGator, DIANAmiRGen v3.0, PASmiR, PhenomiR, DIANATarBase, mimiRNA, miR2Disease, starBase, and Tools4miRs
Algorithms for prediction of precursor miRNA sequences	Triplet-SVM, microPred, MiPred, miPred, miR-BAG, ViralmiR, MiRFinder, and PMirP
Algorithms for the prediction of mature miRNAs	MaturePred, MatureBayes, MiRFinder, miRDup, miRLocator, MatPred, miRanalyzer, and MiRmat

The table was constructed based on publication by Monga and colleagues.^118^

## EV Multi-Omics to Understand Mechanisms of Therapeutic Activity in AKI

EV cargo and function are highly heterogeneous and vary due to both biological and methodological factors. As such, there is considerable interest in profiling EV cargo of different cells and relating this to their observed effects in AKI and other diseases. This not only improves our understanding of AKI pathophysiology but also allows us to identify new potential points for therapeutic intervention. As illustrated in Figure 1, the composition of EVs is complex and diverse, including surface receptors, membrane proteins, soluble proteins, lipids, ribonucleic acids (mRNA, miRNA, tRNA, rRNA, sRNA, snRNA, scnRNA, piRNA, scaRNA, viral RNA, Y RNA, and long noncoding RNA), and DNA. Advances in high-throughput technologies have led to an abundance of data, necessitating the development of bioinformatics tools and databases such as Vesiclepedia and EV-TRACK. These databases aim to standardize reporting parameters and improve reproducibility of studies.^82–84^ As described earlier, experimental variables strongly influence EV cargo and function; thus, these database entries should be interpreted in the context of pre-analytical parameters.^85^

### Publication Trends in EV Omics Studies

**Figure 2.**
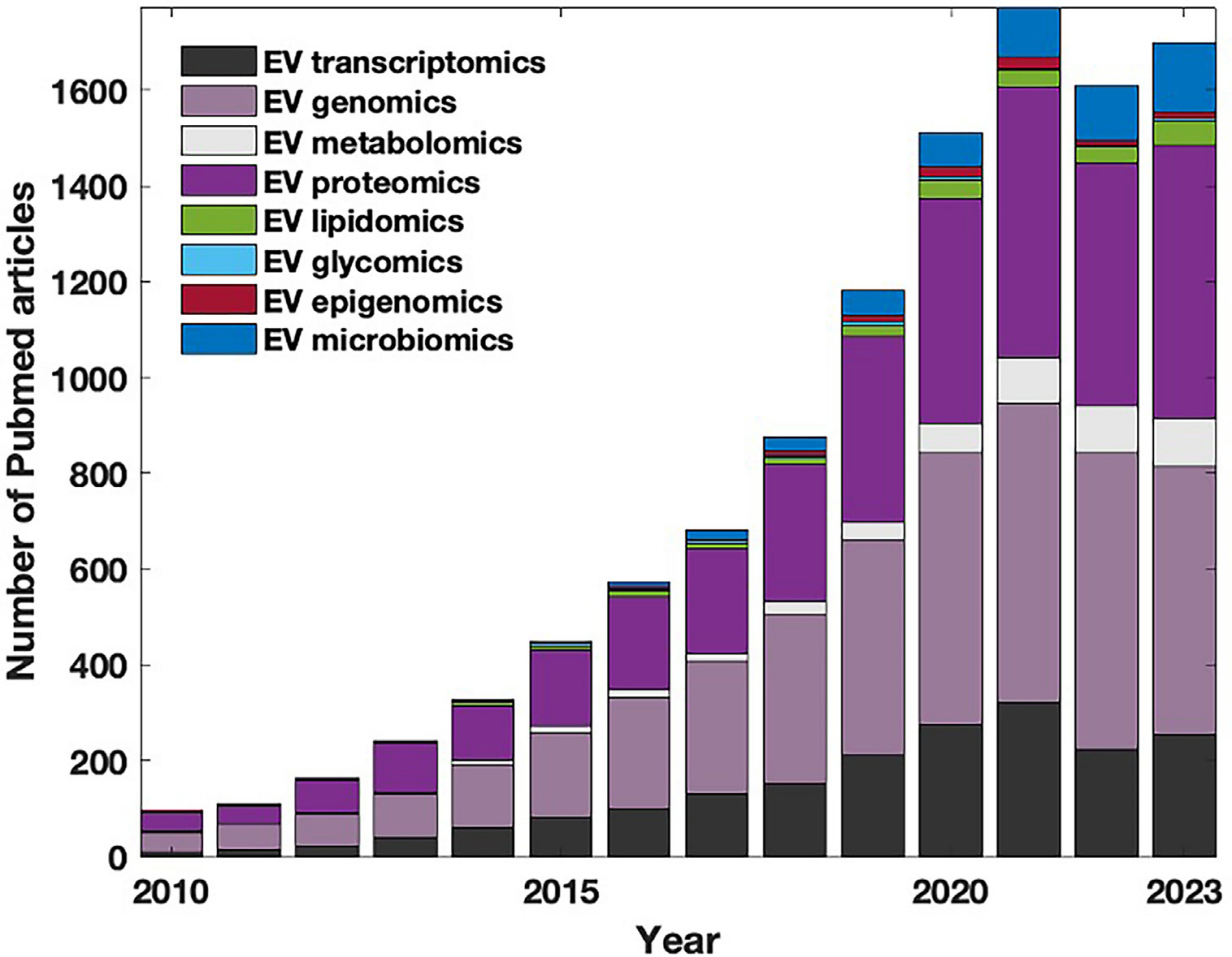
Number of PubMed articles in different EV omics fields, accessed in January 2024. Stacked histogram created with MATLAB 2022Ra. The search was performed using keywords, as shown in the legend.

An analysis of publication trends (Figure 2) demonstrates a steady increase in the number of omics studies for EVs. The top 3 areas are EV genomics, EV proteomics, and EV transcriptomics, comprising 81.4% of EV omics articles on Pubmed in 2023. Newer omics fields, such as EV lipidomics and EV glycomics, have relatively fewer publications than other disciplines. EV lipidomics is an emerging field that appears to be of great importance, since EV lipid composition affects both physical (size, charge, and rigidity) and biological (binding, uptake, and cargo delivery) properties of EVs. Generally, EVs are enriched in glycosphingolipids, sphingomyelins, phosphatidylethanolamines, phosphatidylserines, phosphatidylcholines, and cholesterol compared to the plasma membranes of their originating cells.^74^ EV lipidomics may receive less research attention due to methodological challenges, including limited working sample sizes, which require high-sensitivity mass spectrometry to detect constituents of the EV lipidome. Additionally, effectively separating many lipids remains problematic.^86^ Similarly, the relative lack of glycomics studies is related to challenges in identification of complex carbohydrate structures and the lack of sensitive and high-throughput methods for glycan analysis.^82^ EV microbiomics holds a lot of potential, especially in relation to intestinal microbiota-derived membrane vesicles, as a promising therapeutic tool for chronic kidney disease.^87^

### EV miRNAs in AKI

miRNAs are short (∼22 nucleotide) non-coding RNAs that can bind to complementary sequences in mRNA, targeting them for degradation and reducing translation of specific proteins. Since miRNAs can bind multiple mRNAs, they can simultaneously modulate several cellular pathways, affecting processes such as apoptosis, cell cycle, differentiation, or metabolism. Given that most extracellular miRNAs appear to be protected inside vesicles, EV miRNAs have received considerable research interest.^88^ EV miRNAs can be studied by high-throughput transcriptomic methods such as microarray, RNA-seq, qRT-PCR, and deep sequencing. ^89–91^ PCR-based methods, including large panels such as the Qiagen miRNome system, have high specificity and sensitivity and require relatively low amounts of starting material, hence their widespread use. However, one limitation is that these defined panels can only detect known sequences and may bias cargo characterization towards known common miRNAs.

An isolated population of EVs may contain hundreds of different miRNAs, which, based on their sequences, are theoretically capable of targeting thousands of genes.^20^ However, determining the true biological significance of a particular miRNA within the context of the EV cargo is challenging, particularly when comparing between different experimental designs. For example, miR-21-5p appears to be ubiquitous across multiple sources of EVs. Studies have attributed pro-inflammatory, anti-inflammatory, pro-fibrotic, anti-fibrotic, pro-apoptotic, and anti-apoptotic functions to miR-21-5p, and it has also been proposed as a biomarker for cancer, cardiovascular disease, lupus, diabetes, neurodegeneration, and more.^92^ In AKI specifically, miR-21 has been shown to reduce renal epithelial cell apoptosis and modulate the immune response following kidney I/R.^93^ miR-21 delivered by endothelial progenitor cell EVs has also been shown to protect sepsis-induced AKI in rats, via the RUNX1 pathway.^94^ This illustrates how a single miRNA can take on different roles, producing different effects depending on the experimental models used for analysis. Given that an EV population can contain hundreds of miRNAs, along with proteins, lipids, metabolites, and other bioactive cargo, the advantages of a holistic multi-omic approach are clear.

To encourage such systematic approaches, databases have been developed including EVmiRNA, for miRNA disease/tissue-specific expression profiles, miREV, a resource to find reference transcripts in studies with comparable experimental setups, and a cell-specific reference dataset for miRNA profiling in human peripheral blood.^95,96^ These databases allow for systematic comparisons of miRNA profiles across EVs from different origins, disease states, and methodologies. However, there are still several uncertainties regarding the biological roles of miRNAs in EV cargo and their relevance to observed functions in AKI. Many studies profile EV miRNAs, then use transfection, mimics, and/or miRNA inhibition to confirm the activity of selected miRNAs, often demonstrating that a specific miRNA can recapitulate some, or all, of the effects of the parent EV. These data are sometimes supported by direct evidence of miRNA binding to specific target sequences using reporters.

Illustrative studies of cell culture-derived EV therapies along with posited bioactive miRNAs are summarized in Table 1. This exemplifies that studies of the same type of cell/EV can identify different causative miRNAs, different downstream activities, and sometimes different activities for the same miRNA. In 1 study, MSC-EVs prevented mitochondrial damage and supported ATP generation in proximal TECs during mouse kidney I/R injury. The authors linked these effects to the presence of miR-200a-3p in the miRNA cargo and showed that miR-200a-3p inhibition reduced the therapeutic benefits of MSC-EVs.^80^ Another study with a similar experimental design also showed that BM-MSC EVs protected mouse kidneys from I/R injury. However, the observed therapeutic effects were attributed to the reduction of endoplasmic reticulum stress by miR-199a-3p in the MSC-EV cargo, which was supported by experimental evidence using both miR-199a overexpression and inhibition, which improved and reduced therapeutic efficacy respectively.^97^ In 2 comprehensive studies, cord blood cell-derived EVs reduced proximal tubule epithelial cell apoptosis following kidney I/R injury.^98,99^ This was linked to the abundant presence of miR-486-5p in the EVs and was experimentally supported by use of miR-486 mimics, which reproduced the same therapeutic benefits as the whole EVs. In another study, MSC-EVs were again found to protect I/R-injured kidneys, which was linked to EV miR-125b, comprising 6.8% of the total miRNA cargo. Inhibition of miR-125b removed the protective benefits of MSC-EVs while miR-125b mimics protected hypoxic HK-2 cells.^100^ These studies all align in demonstrating overall therapeutic value of EVs in AKI; however, it is noteworthy that each study identified different essential miRNAs during kidney I/R therapy. This suggests considerable heterogeneity, either in the MSC/MSC-EV phenotype and miRNA cargo, or methodological variations between studies. In addition, online databases such as Vesiclepedia and EV-TRACK show that the above-mentioned miRNAs have all been previously detected in MSC-EVs and many other types of EV, including plasma and serum EVs. Thus, we believe that the concept of EV therapeutic benefits being driven by the presence of single essential miRNAs within the EV cargo is likely an oversimplification and should be approached with caution. Since an EV population contains anywhere from 100 to 1000 different miRNAs in varying quantities, we must consider that any active miRNA is also delivered alongside many others, which may have supportive, synergistic, neutral, or opposing effects on recipient cells. If an MSC-EV contains miR-21-5p, miR-200a, miR-125b, and miR-486, then it would be assumed to work on all those associated pathways. However, some EV cargo miRNAs have been posited to have detrimental effects in AKI models. For example, miR-19b in TEC-EVs was shown to polarize macrophages, through SOCS-1 inhibition, to a more pro-inflammatory “M1” phenotype following LPS-induced AKI, resulting in poorer outcomes.^101^ This is in contrast to a myocardial infarction model where miR-19a/miR-19b was shown as strongly protective by decreasing apoptosis by BIM/PTEN binding, decreasing macrophage M1 polarization, and increasing M2 polarization.^102^ This resulted in anti-inflammatory activities, which were the opposite of the effects found in the kidney. Again, this indicates that the same miRNA may have different functions in different injury models. Thus, if an EV is found to contain both miR-21 and miR-19, it is difficult to predict whether it will have beneficial or detrimental effects. It is also likely that interactions with other EV cargo (other miRNAs or proteins, lipids, metabolites, etc.) play a role. In the context of AKI, a population of MSC-EVs delivered to the kidney would be taken up by multiple cell types, including TECs, macrophages, and endothelial cells, as well as circulating immune cells and other body cells. A systems biology perspective, emphasizing the collective action of multiple miRNAs within a population of EVs may provide a more holistic understanding of their therapeutic potential and mechanisms of action. This is particularly valuable if it can be combined with analysis of gene expression changes in target tissues or combined with EV proteomics or other omics disciplines. Relevant bioinformatic tools used for miRNA analysis and target prediction are shown in Table 2. To quantitatively characterize multi-input miRNA sensors, which may have multiple target sites, with the potential for cooperative effects across them, antagonistic/synergistic computational models using multi-input miRNA classifiers have been developed to test best classifier candidates in cells.^103,104^ Such models allow for simulating a range of classifier designs *in silico*, thus testing only the best candidates experimentally. Taken together, miRNAs contained in EVs clearly play roles in endogenous disease processes, and they can be harnessed for therapeutic purposes in AKI. However, there are still many answered questions regarding their abundance, relative importance, and interactions with other constituents of the EV cargo.

**Table 2.** Selected studies of cell culture-derived EVs in AKI models, focusing on MSC-EVs.

EV	Model	Dose	Findings	Mechanism	Ref.
BM-MSC or HDF, UC	SCID mice Glycerol-induced AKI	15 µg EVs, 75 000 BM-MSC or HDF per mouse, D3 post-AKI, by IV	Equal efficacy to 75 000 MSCs. Increased distal and proximal TEC proliferation. Increased mitochondria. Reduced apoptosis. Reduced BUN/CRE.	RNA transfer.	^160^
BM-MSC or HDF	SD rat, monolateral nephrectomy, 45 m I/R on remaining kidney	30 µg EVs per rat immediately post I/R	BM-MSC EVs reduced CRE to normal levels and lowered BUN. Lower apoptosis. Less scarring at 6 month follow-up. HDF EVs had no effect.	EVs found in I/R kidney within 2 h and 6 h. No longer detectable at 24 h. BM-MSC EV benefits removed by RNAse.	^159^
EPC-EV, UC	Wistar rat, nephrectomy and unilateral I/R 45 m	30 µg EVs immediately post I/R	EPC-EVs lowered CRE to normal levels and reduced BUN. Improved renal histology. Increased proliferation (BrdU), reduced apoptosis (TUNEL), and reduced immune cell infiltration. A 6 month follow-up showed prolonged benefits. HDF-EVs had no therapeutic effect.	RNAse treatment rendered EPC-EVs ineffective. miR-126 and miR-296 antagomiR treatment of EPCs reduced EV therapeutic effects. EPC-EVs also lowered tubular endothelial cell inflammation and enhanced tube formation under hypoxia.	^170^
BM-MSC or HDF, UC	SCID mice, 12 mg/kg SC cisplatin (lethal in 5 d)	100 µg EVs by IV, 8 h (single dose) or 8 h, 2 d, 6 d, 10 d, 14 d, and 18 d(multi-dose) post-Cisplatin	Increased survival at d21 (40% single dose and 80% multi dose). Reduced necrosis, BUN/CRE, and TEC apoptosis. No benefit from fibroblast EVs.	RNA transfer. Expression on human proteins in kidney. RNAse treatment removed all therapeutic benefits.	^196^
Umbilical MSC, UC	SD rat, unilateral I/R for 60 m	30 µg EV per rat immediately post I/R	TEC de-differentiation (vimentin) and proliferation (PCNA) at 48 h. Increased rat HGF expression. Benefits prevented by c-MET and MEK inhibitors.	RNAse treated hUC-MSC EVs had no effect. Induction of rat HGF expression and human HGF by mRNA transfer.	^166^
Mouse BM-MSC, co-culture	KM/NIH mouse, bilateral I/R 60 m	2 × 10^6^ cells by IV	Reduced apoptosis in kidney sections. Lower CRE/BUN than I/R mice.	No specific EV isolation, but co-culture transferred miR-233. Luciferase showed miR-233 inhibits NLRP3. miR-233 inhibition reduced efficacy of MSC therapy and lowered HGF, IGF-1 and VEGF production.	^162^
Umbilical MSC, UC	C57/BL6 mice, bilateral I/R 30 m	50 µg or 100 µg EVs per mouse, by IV, 0 and 24 h post-I/R	EV localization to proximal tubules. Approximate 2.5-fold increase in EV uptake by I/R kidney compared to sham. Dose-dependent reduction in CRE. Reduction in Kim-1^+^ tubules. Reduced apoptosis (TUNEL). Increased TEC proliferation.	Homing to the I/R kidney by VLA-4 and LFA-1 binding. miRNA quantification (by qPCR) found miR-100, miR-26a, and miR-125b. miR-125b inhibitors reduced protective effects of EVs. miR-125b was shown to bind and inhibit p53	^100^
Placental MSC, UC	FVB mice, unilateral I/R 50 m	80 µg per mouse, by IV	Increased uptake by I/R kidney 2 h and 24 h post-I/R. Reduced BUN/CRE at 3d, 5d, and 8d post-I/R. Reduced Kim-1 + tubules and necrosis. Protection of mitochondrial number and function.	EVs modulated expression of Keap1, Nrf2 and Sod2, and lowered ROS. EV miR-200a-3p recapitulated these same effects by targeting Keap1. miR-200a inhibition reduced protective effects.	^80^
BM-MSC (species not stated)	BALB/c mice, I/R 45 m	5 × 10^10^ particles per mouse, IV, 1 h before I/R	Small (10-20%) reductions in CRE and urea at 24 h post-I/R. Reduced apoptosis and improved kidney morphology.	Apoptotic protein BIP was targeted by EV miR-199a, confirmed by luciferase, miRNA transfection, and inhibition in NRK-52E *in vitro* model.	^97^
BM-MSC, UC	BALB/c mice, bilateral I/R 45 m	5 × 10^10^ particles per mouse, IV, 1 h before I/R	Reduced TEC apoptosis, reduced serum BUN/CRE, and improved morphology.	EV miR-199a was shown to downregulate Sema3A and activate ERK/akt pathways, reducing apoptosis. This was confirmed using miR-199 mimics and inhibitors, which increased and decreased HK-2 cells.	^163^
Autologous ad-MSC, UC	Domestic pigs, metabolic syndrome (high carb and high fat diet) with renal stenosis induced by arterial irritant coil	1 × 10^11^ EVs or 1 × 10^7^ MSCs per pig (50-60 kg), 6 weeks after renal artery stenosis injury	EVs and MSCs reduced apoptosis of endothelial, epithelial, and interstitial cells. Both reduced fibrosis and fibrotic gene expression. MSCs (but not EVs), restored microvessel density, whereas EVs reduced necroptosis.	Both EVs and MSCs improved morphological and functional parameters. The MSC-EV group showed increased miR-532-5p expression compared to MSCs. MSCs had stronger anti-inflammatory effects than MSC-EVs.	^67^
BM-MSC, UC (211 nm)	SD rat, nephrectomy with I/R on remaining kidney for 45 m. Some animals received simultaneous splenectomy.	100 µg EVs per rat, IV. Sacrifice at 24 h	EVs reduced CRE/BUN at 24 h. Lower tubular injury (histology). NK cell inhibition (by anti-CD161 mAb) also improved parameters.	Splenectomy did not affect MSC-EV protective effects, but did reduce NK cell recruitment to I/R kidney, corresponding with TLR-2 and CX3CL1 downregulation.	^164^
ECFC, UC	FVB mice, bilateral I/R 30 m	20 µg EVs per mouse, IV, at reperfusion	EVs reduced PTEN, activated AKT, and altered genes associated with metabolism. In mice, EVs reduced CRE/BUN, improved kidney morphology, and reduced apoptosis (TUNEL). In *in vitro* models, ECFC-EVs stimulated HUVEC tube formation.	EVs were rich in miR-486-5p. miR-486 mimics also reduced PTEN, activated AKT, and reduced tubule apoptosis. miR-486 inhibition reduced almost all therapeutic effects of the ECFC-EVs.	^98,99^

Where papers include both AKI and CKD components, we describe AKI. We also described primarily *in vivo* results. All cells are human, unless stated otherwise. EPC, endothelial progenitor cells; ECFC, cord blood endothelial colony-forming cell; BM, bone marrow; MSC, mesenchymal stromal/stem cell; IV, intravenous; SC, subcutaneous; BUN, blood urea nitrogen; CRE, creatinine; TEC, tubular epithelial cell; UC, ultracentrifugation; hUC, human umbilical cord; HDF, human dermal fibroblasts; and ROS, reactive oxygen species.

Further complicating the understanding of EV miRNAs, a comprehensive study by Chevillet and colleagues demonstrated that even the most abundant miRNAs in a given EV population were present at a rate of only 1 copy per 100 EVs.^105^ Their study indicates that the majority of EVs, whether from cultured cells or body fluids, were absent of any miRNAs, which was consistent across multiple methodological approaches of EV isolation and miRNA measurements. Since it is known that EVs deliver ribonucleic acids very efficiently, this does not rule out their biological activity or importance. However, the relative rarity of miRNAs does challenge the premise that they are the predominant functional component of EVs or the biological reason for EV secretion. This may explain some of the apparent discrepancies or inconsistencies in the field. We suggest that if miRNAs are the supposed mechanism of EV activity in an experimental design, the miRNA copy number could be considered as part of the determination of EV dose. For example, if the EV particle number and miRNA copy number of a particulate isolate are known, the number of miRNAs delivered per target cell could also be approximated.

### EV Proteomics in AKI

Mass spectrometry (MS)- or western blot-based platforms allowed the identification of thousands of EV-associated proteins, thus making them the most studied EV cargo. MS is a powerful technique ie used to identify unknown compounds (proteins and peptides) and to quantify known ones through a variety of methods, such as electrospray ionization-mass spectrometry (ESI-MS) or matrix-assisted laser desorption ionization-mass spectrometry (MALDI-MS). These methods allow quantification of mass of a compound, or identification of a protein through peptide mass fingerprinting (PMF), using peptide mixtures obtained by digesting unknown proteins with endoproteases. Tandem mass spectrometry (MS/MS) is a key technique based on amino acid sequencing of proteins, peptides, and their post-translational modifications (PTMs), using bottom-up and top-down proteomics analysis.^106^ Bottom-up proteomics can be used for the identification of peptides, proteins, and their PTMs, as well as quantitative proteomics, but is not sufficient for protein structure and function analysis. Top-down approaches analyze intact proteins without prior digestion of proteins into peptides and can provide a wealth of information on the structure of that protein.^107^ Protein microarrays or protein chips are parallel assay systems that contain small amounts of proteins at high densities.^108^ There are 3 main types of protein microarrays based on their reaction principle: analytical or antibody, functional, and reverse-phase microarrays. Analytical microarrays, in which antibodies are arrayed on glass surfaces at high densities, are one of the most powerful multiplexed detection technologies, used for biomarker identification, clinical diagnosis, or food safety analysis.^109^ Functional microarrays enable studying protein interactions, such as protein binding and enzyme-substrate reactions. Reverse-phase microarrays allow for the analysis of many samples by spotting tissue/cell lysates (or fractionated lysates) on glass surfaces.^110^

Vesiclepedia lists the top 100 identified EV proteins by pooling data from hundreds of studies. The most common EV proteins include those from the cell plasma membrane (CD63, CD9, CD81, etc.) and those involved in EV biogenesis (ALIX, TSG101, FLOT1, etc.), all of which are commonly used as markers to confirm EV isolation. To put their abundance in context, a study using quantum dots for 3D super-resolution imaging found an average of 12.6 copies of CD63, 1.6 copies of CD81, and 16.6 copies of CD9 per single seminal EV.^111^ This would make these proteins far more abundant per EV than miRNAs, indicating that they likely have biological function. CD81 in particular has been widely studied and revealed to have multiple functions, including integrin binding, CD19 and CD4/CD8 binding (thus immune cell interaction), and may have anti-fibrotic and anti-inflammatory properties.^112^ It should also be noted that metabolic enzymes such as GAPDH, PKM, and PGK1, as well as ubiquitous proteins such as beta-actin and albumin, are also within the top 25 most detected proteins in EV proteomic studies. Whether these are true constituents of the EV cargo, part of an EV-associated corona, or simply co-isolates, is unclear.

A review of the kidney proteome has been recently published, which highlighted that most proteomic and multi-omic studies in the nephrology field have focused on biomarkers.^113^ Similarly, a recent review of EV proteomics in the context of kidney disease highlighted that most published studies relate to urinary EVs as biomarkers of kidney injury rather than their exploration as therapeutics.^114^ Models of I/R AKI have shown roles for proteins such as AQP1, fetuin A, and NGAL, which are found in EVs secreted from proximal TECs. Again, these can be detected in altered concentrations in the urine following AKI.^22^ As described in the previous section, most MSC-EV/AKI studies focus on miRNAs as drivers of therapeutic benefits; however, MSC-EV preparations have been shown to contain peptide growth factors, such as VEGF, IGF-1, HGF, IL-10, and FGF2, albeit in low concentrations.^84,115^ None of these growth factors appear within the top 100 proteins in the Vesiclepedia proteomic database, but they can be detected using more sensitive methods such as ELISA. IGF-1 can act through the Nrf2/ARE and PI3K/AKT/mTOR pathways to reduce renal TEC apoptosis, and act via the MAPK pathway to increase cell proliferation. FGF-2 has anti-fibrotic functions; IL-10 inhibits inflammation and polarizes macrophages towards an M2 phenotype; and VEGF stimulates microvessel formation.^116,117^

Disease-specific proteome alterations in a range of pathologic states have been also demonstrated, including kidney disease and kidney transplantation.^91,118^ To fully understand EV proteomics, additional layers of protein regulation, such as post-translational modifications (phosphorylation and glycosylation) should be considered, since they can modulate protein structure and function by changing its physicochemical characteristics and interaction partners.^119^ Omics analyses of EVs derived from a single EV subtype may yield more targeted results than bulk approaches.^120^ Comparative proteomic analysis can also identify different EV subtypes from a single cell type.^121^ These technologies may be useful in addressing the diversity of overall EV functions, which may be explained by subpopulations of EVs with different cargo.^122^

### EV DNA

Despite the growing interest in different EV omics sciences, the characterization of EV DNA is still difficult, mainly due to a poor understanding of its functional significance and a lack of standardization in EV analysis techniques.^123^ Even so, the main use of EV DNA is for biomarker development, since EV DNA reflects the parent cell gDNA both qualitatively and quantitatively.^124–126^ Therefore, the characterization of EV DNA could be preferred to the analysis of circulating DNA and EV-derived RNA, since these biomarkers are highly unstable.^127^ Indeed, the potential of EV DNA as a biomarker for kidney allograft injury has been shown by Sedej and colleagues, as several EV DNA characteristics are correlated with clinical and histological parameters.^128^

### EV Multi-Omics and the Role of Machine Learning in AKI

**Figure 3.**
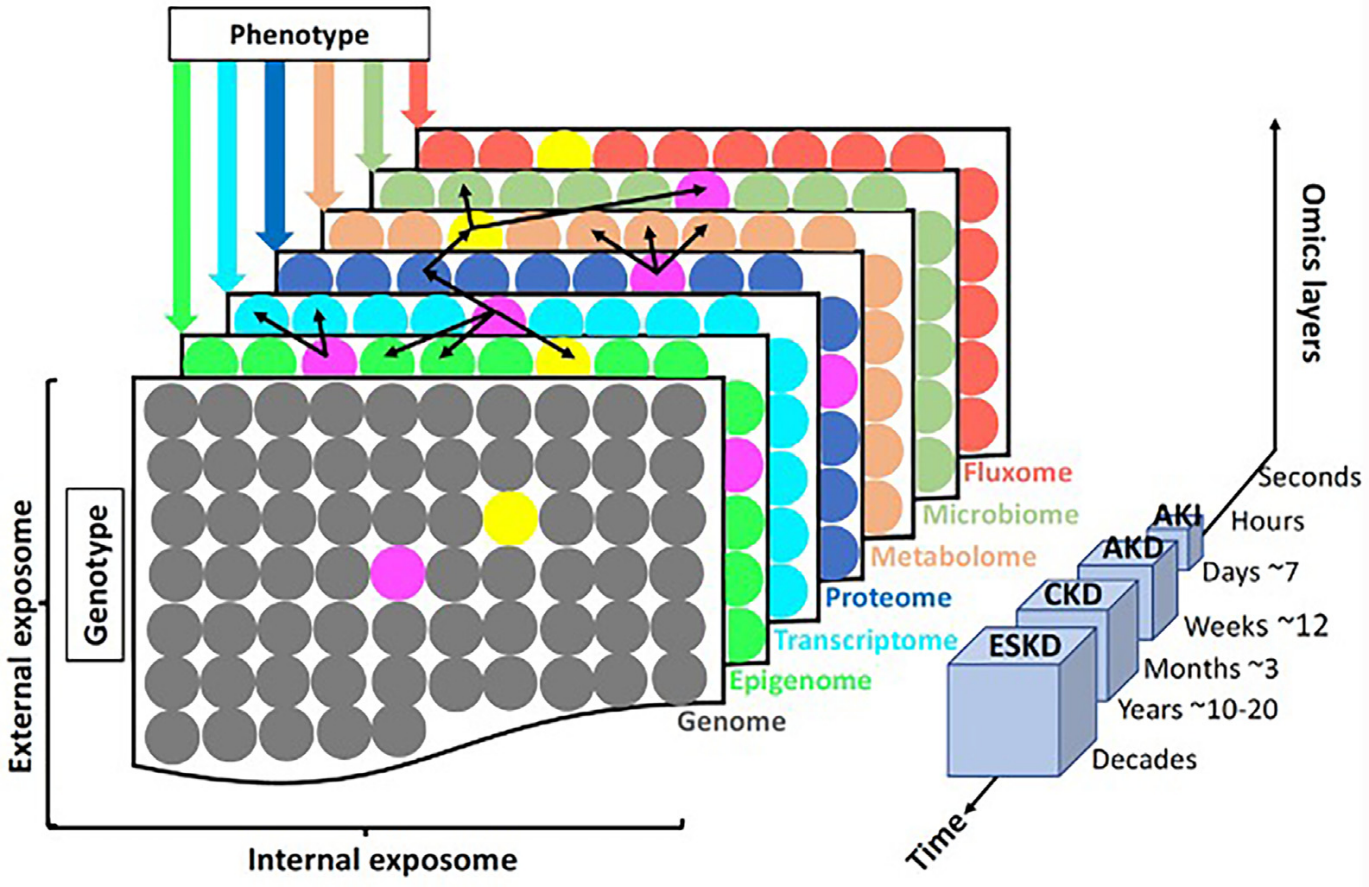
Multi-omics approaches to AKI. Layers depict different types of omics data that characterize individual phenotypes and genotypes. Molecules are represented as circles—eg the yellow transcript can be correlated to multiple proteins. Black arrows indicate potential interactions or correlations between molecules in distinct omics layers that are often changing at different chronological scales, which are linked to kidney disease progression. The internal exposome focuses on biological molecules that are analyzed using omics technologies. The external exposome relies on exposures due to environmental, social, and lifestyle factors. AKI = acute kidney injury; AKD = acute kidney disease; CKD = chronic kidney disease; and ESKD = end stage kidney disease. Staging is based on Levey 2022.^137^

Compared to single omics, multi-omics can provide researchers with a greater understanding of the information flow across different omics layers, from the cause of disease to the functional interactions.^129^ This concept is illustrated in Figure 3. Omics technologies focusing on genomic organization (eg ATAC-seq), gene expression (eg mRNA sequencing), and protein products (eg MS) and their single-cell applications, have greatly improved molecular insights into AKI. Due to the multi-factorial nature of AKI, these and other multi-omics approaches will advance our understanding of kidney injury and its transition from acute to chronic stage.^130^ Multi-omics approaches shed new insights into the pathogenesis of cisplatin-induced AKI, using transcriptome (mRNA expression), proteome, and N-degradome analyses.^131^ The 3 omics layers, acquired from the same kidney cortex of the same mouse, revealed a weak correlation between the transcriptome and proteome. Interestingly, this discrepancy was weaker in kidneys that were functionally impaired, likely due to changes in the complement system, as supported by N-degradome analysis. Such multi-omic investigations might provide valuable insights into the pathophysiology of AKI, as protease activity can be detected at system’s level, which cannot be observed with transcriptomic analyses alone.

The dysregulation of another important EV cargo, miRNAs, which are critical regulators of cellular homeostasis, could contribute to kidney pathophysiology. To delineate the tissue-specific characterization of miRNA-target interactions, different web applications have been developed, such as databases of validated miRNA-target interactions and prediction tools. For example, IMOTA (interactive multi-omics tissue atlas) that includes over 23 000 relations between miRNAs and 23 human tissues, and over 310 000 relations between mRNAs and the same tissues.^132^ Such bioinformatics services are also available for other classes of non-coding RNAs, eg competitive endogenous RNA (ceRNA). miRTissue_ce_ is a powerful tool for the analysis and characterization of ceRNA-miRNA-mRNA crosstalk across the transcriptome in different tissue types, thus improving the understanding of more complex regulatory mechanisms.^133^

Searches in PubMed for the term “EV multiomics OR EV multi-omics” returned 128 hits after 2019, with growing numbers over the last 5 years (as of February 2024). Narrowing this search for “kidney” or “renal” returned only 7 hits. Although the number of these studies is relatively small, multi-omics approaches applied to EVs in kidney disease have been successfully utilized in cardiorenal syndrome, renal cell carcinoma, and inflammatory kidney disease.^134–136^

Increasing evidence indicates that EV-mediated cell-to-cell communication can be specific, rather than generic, and can be investigated by multi-omics.^123^ For example, proteomics of tumor-derived EVs revealed that EVs could prepare the pre-metastatic niche in an organ-specific manner.^77^ EVs can be viewed as molecular communication systems, whose theoretical aspects of EV delivery have been extensively studied using sophisticated mathematical modeling.^138^ Since integration strategies of multi-omics data have been mainly focused on biomarker discovery, not all methodologies are applicable to EV studies. However, the 2 most translatable integration methods for EV studies are correlation-based methods (CBM) and network-based methods (NBM).^139^ CBM can be used to investigate the relationship between 2 omics datasets, although it does not account for correlations between indirectly associated omics data.^140^ The advantage of NBM is the resemblance to the interconnectivity of a biological system, where nodes could represent biomolecules of the EV cargo, and edges would define interactions between them, which can be inferred based on previous knowledge, such as ontologies.^139^ Nevertheless, there are still many improvements required, such as the standardization of sample collection and processing, transparent reporting of experimental design for reproducibility, and finally, availability and consistency of data.^140^ Once these obstacles in a single omics layer are overcome, the integration of different omics layers also has its challenges. In particular, addressing variables (eg age, sex, BMI, lifestyle, etc.), which may affect the properties of patient-derived EVs, should be considered using advanced statistical methods.^121,141^ The numerous issues arising in this field highlight the urgent need of reproducibility and standardization at all levels of EV multi-omic analysis, and the importance of novel computational/bioinformatics tools.

Since AKI complicates 13-18% of hospital admissions, artificial intelligence (AI) in AKI research has focused mainly on risk prediction from risk scores to automated electronic alerts.^142,143^ Most models are developed in a single healthcare system (eg using electronic health records) and often lack external validation, which is necessary to assess the generalizability and performance of AI models.^144–146^ One study performed external validation using a gradient-boosted machine learning (ML) model that demonstrated excellent discrimination in both internal and external validation, supporting its potential as a clinical decision support tool for AKI detection.^147^ Using mRNA or miRNA expression levels, ML algorithms have been employed to identify characteristic genes in AKI, predict potential drugs for AKI, and analyze molecular responses to ischemia/reperfusion injury (IRI) during the AKI-to-CKI transition.^148–150^ An overview of ML methods in miRNA discovery and target predictions can be found in a review by Parveen and colleagues.^151^

The combination of AI and omics will assist with translating multilevel data into clinical practice.^152^ In the future, omics input will guide therapies and clinical decision-making, which will require correct interpretation through integration with clinical data and parallel advancements in technical infrastructure. Omics combined with AI have already been applied to EVs in subtypes of renal carcinoma and ESKD.^153–155^ Furthermore, multi-omics for predicting drug-induced kidney injury will likely become a central topic.^156^ Increased interest from the industry to save drug development costs will require computational strategies for drug repurposing, a cost-effective way of developing new targets for existing drugs.^157^ Fortunately, the kidney disease field can utilize approaches from other fields, such as oncology. The progress in AI and omics will open new frontiers that may revolutionize renal medicine.

## Mesenchymal Stromal Cell-Derived EVs in AKI

### Studies of MSCs and MSC-EVs in AKI

As mentioned in previous sections and summarized in Table 1, MSCs and MSC-EVs have been extensively studied for their therapeutic potential in the treatment of AKI and their efficacy is well supported. Multiple human clinical trials have examined the use of MSCs as a cell therapy in kidney diseases, as reviewed by Liu et. al.^158^ Most of these trials have been conducted in the setting of CKD rather than AKI, and there is a wide variety in the MSC dose (from 1 × 10^5^ to 2 × 10^8^ cells/kg), administration route (intravenous, intra-arterial, or intra-aortic), and cell source (autologous or allogeneic and adipose-derived or bone marrow-derived cells). Overall, the published results of these trials are encouraging, with MSCs showing little cause for concern regarding safety. Efficacy varies, with some trials showing no detectable effects and others showing mild improvements in estimated glomerular filtration rate (eGFR), serum urea, and circulating inflammatory markers. Section 2.5 previously mentioned benefits of EVs compared to cell therapy. The first reported use of MSC-EVs in AKI therapy (in an animal model) was published by ^159^ and colleagues in 2009. Bone marrow MSC-EVs were shown to increase proliferation and reduce apoptosis of TECs following glycerol-induced AKI.^160^ Multiple studies across multiple animal models of AKI have shown similar findings.^161^ A wide variety of beneficial effects including reduced TEC apoptosis, increased TEC proliferation, reprograming, increased renal anti-oxidative capacity, increased angiogenesis, immunosuppression, and anti-inflammation have all been reported. As illustrated in Section 3, there is great diversity in the reported “active” substances across these studies; EV-mediated delivery of miR-148b, miR-410, miR-495, miR-223, miR-30, miR-199, miR-125a, miR-15a, and many others have all been reported as essential for observed therapeutic effects of MSC-EVs.^97^,^162–164^ This again illustrates the need for integrated multi-omic approaches.

Here, we highlight some of the most interesting findings from selected studies in Table 1. Overall results consistently show that MSC-EVs produce benefits in multiple AKI models, including unilateral or bilateral I/R, cisplatin toxicity, and intramuscular glycerol (as a model of rhabdomyolysis). This is validated by standard kidney function markers (serum creatinine and urea) and histological analyses. EVs also appear to act rapidly, with some experiments showing favorable improvements after time points as short as 2 h post-injury. The dose, administration route, and timing vary significantly between studies. For example, studies featured in Table 1 have used single EV doses ranging from 30 µg per rat to 100 µg per mouse, or 5 × 10^10^ EVs per (25-30 g) mouse to 1 × 10^11^ per (50-60 kg) pig.

Although miRNAs are the most commonly investigated type of EV cargo, they are not the only therapeutic component. One interesting study reported that MSC-EV membranes contain CCR2, which was able to bind free CCL2, thereby lowering inflammatory signaling following I/R-induced AKI.^165^ This was supported by experiments showing that CCR2 overexpression could reduce free CCL2 levels, whereas CCR2 knockdown reduced therapeutic effects of MSC-EVs. This concept of EVs acting as decoy receptors to soak up cytokines is a different paradigm the typical cargo delivery-based model, since it does not rely on EV uptake, nor does it require altering recipient cell protein expression, thus it may act more quickly. Another alternative paradigm is that EV therapy may be mediated by mRNA transfer. For example, 1 study of AKI claimed that MSC-EVs contained (human) HGF mRNA, which could be transferred into rat TECs following AKI, resulting in transcription and translation of human HGF in the rat kidney. Therapeutic benefits could be counteracted by RNAse treatment or small-molecule-based inhibition of HGF signaling.^166^ These concepts are also illustrated in Figure 1.

Interestingly, several studies featured in Table 1 used human dermal fibroblast (HDF)-derived EVs as a control, and described that they lacked significant protective effects in AKI models. Grange and colleagues showed that MSC-EVs, but not fibroblast EVs, reduced fibrosis, increased urine output, and improved serum biomarkers following diabetic nephropathy in mice.^167^

Interestingly, EVs from HDFs have been shown to contain miRNAs such as miR-21-5p, miR-125b, and miR-199 (Vesiclepedia_3456), which have been described as protective in AKI.^168,169^ Therefore, it is not immediately clear why HDF-EVs do not have therapeutic effects. One possible explanation is that HDF-EVs also contain other cargo ie detrimental or counteracts the benefits of beneficial cargo. This is another area in which a multi-omic approach may be useful to take a holistic view of EV cargo and observed functions. Another possibility is that the HDF-EV surface protein profile does not allow them to deliver cargo into TECs with the same efficiency as MSC-EVs. Supporting this hypothesis, there is evidence that MSC-EVs could bind to injured kidney tissue through CD44 and CD29, while EVs derived from fibroblasts were unable to do so. ^159,163,166^ This indicates that a combination of surface proteins and miRNA cargo is essential for EV therapeutic benefits, and again highlights the value of a multi-omic approach, which could combine miRNA and proteomic analyses. It is also interesting that some studies showed that RNAse treatment removed beneficial effects of MSC-EVs, thus demonstrating that mRNA/miRNA were the therapeutic EV cargo.^160,169,170^ However, other studies have shown that EVs are capable of protecting RNA cargo from degradation by RNAs.^27,68,171^ These discrepancies may be attributed to methodological differences, or due to EV isolations containing remnant extravesicular RNA species.

Taken together, there is abundant evidence from animals showing efficacy of MSC-EVs in AKI models. Unfortunately, there are fewer published clinical trials on MSC-EVs in human kidney disease.^117,161^ One relevant randomized phase II/III study by Nassar et. al. recruited 40 patients with stage III or stage IV CKD and administered umbilical cord-derived MSC-EVs or a placebo control.^172^ The administered EVs were obtained by UC and ranged from 80 to 1000 nm in diameter (mean diameter 435 nm), indicating a more heterogeneous EV population than the ∼100 nm vesicles seen in most research publications. EVs were given at 100 µg EV protein per kg per dose. After 2 intra-arterial doses 1 week apart, patients began to show significant improvements from 8 weeks, with increased eGFR (based on creatinine) and decreased serum urea, which lasted until the end of the trial after 12 months. Patients receiving MSC-EVs also showed significantly higher plasma levels of IL-10 and TGF-β1, and lower levels of plasma TNF-α.

Taken together, multiple studies have demonstrated that MSC-EVs are beneficial following AKI. However, there is great diversity in the reported mechanisms of action, including EV uptake and cargo delivery, mRNA transfer and translation, and EVs forming decoy receptors. There is also diversity in the reported active beneficial components of MSC-EVs and their downstream actions, including macrophage polarization, stimulation of endothelial cell or TEC proliferation, and reducing of TEC apoptosis.

## Non-Stem Cell-Derived EV in AKI

Although MSC-EVs are the most commonly researched, EVs derived from other cell types have shown therapeutic potential in the context of kidney diseases. Here, we overview the role of some types of non-MSC-EVs in the context of AKI. These studies are also featured in Table 1.

### Endothelial Progenitor Cell-Derived EVs

Cantaluppi and colleagues found that endothelial progenitor cell (EPC)-derived EVs could prevent AKI from I/R injury in a rat model, through miR-126 and miR-296-mediated reprograming the hypoxic renal parenchymal cells into a regenerative state.^170^ Zhang and colleagues also found that EPC-derived EVs could alleviate renal tissue inflammation and apoptosis in rat sepsis-induced AKI via upregulating miR-21-5p expression, resulting in runt-related transcription factor 1 (RUNX1) silencing. The effect was recapitulated by miR-21 agomirs and partially reversed by miR-21 inhibition.^94^ Recently, Vinas et al. found that intravenous injection of cord blood endothelial colony-forming cell-derived EVs (enriched in miR-486-5p) could protect against I/R-induced AKI in mice.^98,99^ The underlying mechanisms included modification of renal epithelial cell transcriptomes (proinflammatory and apoptotic pathways) and endothelial cell transcriptomes (metabolic pathways), respectively. miR-486-5p was found to bind PTEN, which was recapitulated by miRNA mimics. On the other hand, treatment of EPCs with anti-miR-486-5p removed the therapeutic benefits of EPC-EVs, demonstrating that miR-486-5p was essential for therapeutic functions. Interestingly, miR-486-5p has also been identified in EVs from human serum, cancer cells, CSF, and others (Vesiclepedia experiment ID 2320, 1033, and 1203, and a previous study from our group ^29^), demonstrating that it is not exclusive to EPC-EVs.

### Epithelial Cell-Derived EVs

Human amniotic epithelial cells (hAEC) and hAEC-EVs have been demonstrated as efficacious in several models of AKI, such as cisplatin-induced AKI, I/R AKI, and sepsis-associated AKI.^66,173,174^ Mechanistically, the renoprotective effects through intravenous delivery of hAEC/hAEC-EVs have been demonstrated via suppression of TNF-α/MAPK and caspase signaling pathways, resulting in reduced tubulointerstitial injury and therefore improving renal function. Notably, EVs derived from rat or human renal TEC, pre-exposed to 1% O_2_ hypoxia for 4 h, mimicking an ischaemic preconditioning state, have been found to exert therapeutic effects on ischemic AKI rats when administered IV.^175,176^ EV treatment improved renal function and diminished renal inflammation, oxidative stress, and subsequent peritubular fibrosis. Interestingly, comparison of RNA sequencing data from injured kidney tissues (EV-treated versus non-treated group) further confirmed that EV treatment substantially corrected the altered transcriptomic profiles found in the non-treated group.^175^ In LPS-induced AKI mice, it has been shown that TEC-derived EV miR-19b-3p could be engulfed by macrophages, leading to M1 macrophage activation and proinflammatory responses *via* the NF-κB/SOCS-1 axis, which was overall detrimental to kidney function.^101^ Hence, the therapeutic advantages or disadvantages of TEC-derived EVs may depend on the state of TEC to exert effector functions through EV cargos.

### Immune Cell-Derived EVs

The dramatic expansion of EV research in the past decade has shown that EVs are involved in both protective and damaging immune responses in health and disease.^177,178^ In fact, it is conceivable that EVs are fundamental inflammatory mediators alongside well-known cytokines/chemokines, participating in intercellular communications. Almost every type of immune cells has been shown to possess the ability to produce EVs, and some of their individual functions have been characterized. Among them, neutrophils, the most abundant leukocytes in blood, can secrete a wide range of EVs, either pro-inflammatory or anti-inflammatory, depending on microenvironmental cues. Of note, EVs derived from neutrophils stimulated with *N*-formylmethionyl-leucyl-phenylalanine could prime resting neutrophil’s activity for NADPH oxidase activity and augment phagocytosis capacity.^179^ In a murine heart transplant model, donor antigen-presenting cells (APC), mainly dendritic cells (DC), secrete EVs containing MHC molecules that could activate alloreactive T cells, potentially contributing to acute graft rejection.^180^ Notably, the therapeutic potential of macrophage-derived exosomes in treating AKI has been addressed very recently. First, M2 polarized macrophage-derived EVs encapsulated IL-10 could target the injured kidney after intravenous administration through several adhesive components (ie integrin α4β1, α5β1, αLβ2, and αMβ2) on the EV surface, thereby ameliorate I/R AKI.^181^ Subsequent studies identified that EVs derived from infiltrated macrophages upon I/R-AKI contain miR-195a-5p and miR-155, which induce renal tubular cell injury and AKI progression.^182,183^ In summary, there is evidence that EVs derived from immune cells or blood cells could contribute to physiological and pathological processes in kidney homeostasis and disease.

### Engineered EVs in AKI Therapy

Many attempts have been made to engineer EVs by gene editing of the originating cells to overexpress certain surface proteins, by directly modifying EV surface proteins, by attaching polymers to the EV surface, or by combining EVs with responsive ligands to improve targeting efficiency. These approaches have been recently reviewed elsewhere.^53,184,185^ On the other hand, synthetic nanocarriers can be fabricated with a good degree of control, and so-called EV-mimetic liposomes incorporating EV surface proteins have been described, again aiming to improve targeting efficiency and cargo delivery.^186^

EV engineering has been used to load EVs with therapeutic proteins for AKI therapy, thereby using EVs as a biologically derived drug delivery vehicle. As mentioned above, IL-10 overexpression in macrophage-derived EVs was more protective of I/R AKI than unmodified EVs from the same cells.^181^ A recent study engineered an NF-kB repressor into EVs, which suppressed inflammation after I/R AKI to a greater degree than naïve EVs.^187^ In an example of specific targeting of the injured kidney, Tang and colleagues showed that in I/R- and UUO-induced kidney injury mice, engineered red blood cell-derived EVs with Kim-1 targeting peptides and delivered siRNAs for repressing transcription factors *P65* and *Snai1*, could alleviate inflammation and fibrosis in the injured renal tubules and promote renal recovery.^188^ Wu and colleagues also demonstrated that hybrid vesicles formed from MSC-EVs and neutrophil membranes had superior uptake by TECs and reduced uptake by macrophages, and were more effective in a model of cisplatin-induced AKI than regular MSC-EVs. Treated mice showed lower BUN/CRE, reduced TEC apoptosis, and lower inflammatory cytokines. The difference in cell-specific uptake was attributed to CD47, which was found on neutrophil membranes but not MSC-EVs. ^189^

## Clinical Perspectives on EV Treatment: Challenges and Opportunities

### Delivery Routes for EV Therapy of AKI

Despite EVs showing great potential in pre-clinical therapeutic models, several hurdles must be overcome before actual benefits on humans can be realized. First, the optimal EV delivery route in humans remains uncertain. Most animal studies use intravenous administration for EV delivery to the kidneys, resulting in relatively low uptake, as described earlier.^81^ As an intravenous therapeutic EVs have poor circulatory properties, since they are rapidly cleared from circulation, with more than 70% removed after only 5 min.^65,81^ Following IV delivery, EVs must travel to the heart, then through pulmonary circulation before they reach systemic circulation, whereupon the kidney receives ∼20% of cardiac output. As such, many EVs are retained by upstream organs, including the lungs and clearance organs such as liver and spleen, and are taken up by peripheral blood mononuclear cells (PBMCs) while in circulation.^81^ Therefore, baseline EV uptake by the kidney is low. Following kidney injury, local vascular permeability is increased, basement membranes are damaged, and EV uptake and retention may be increased by both passive and active mechanisms. For example, studies have shown that there is increased uptake in injured kidneys due to upregulation of adherent intercellular cell adhesion molecule 1 (ICAM1) for promoting cell fusion, as well as increased local chemokines (ie monocyte chemoattractant protein-1 and MCP-1) for attracting EVs bearing corresponding chemokine receptors (such as CCR2).^165,175^ However, this effect is relatively weak and most EVs are still retained by other organs. In humans, the delivery route would be longer, thus the delivered EV dose would be expected to be even lower.

Intra-renal artery injection can also be considered, which is a more complex and invasive procedure, but it can deliver EVs more directly to the injured kidney.^190^ In humans, this would be possible when applying image-guided endovascular techniques with transarterial catheter probing to the orifice of the respective renal artery. A similar technique has been demonstrated with stem cell delivery in a porcine model.^191^ The procedure is invasive and there are some safety concerns with prolonged catheter placement, especially if repetitive injections are needed. However, a major advantage is that established renal vascular networks can achieve a more complete distribution of EVs throughout the structure of the organ. Intra-renal artery was used for a human clinical trial of MSC-EVs and no adverse events were reported.^172^ Alternatively, intraparenchymal administration can be achieved by percutaneous, ultrasound-guided injection, following similar procedures to a kidney biopsy. The procedure itself is relatively fast (under 1 h) and safe, though there is some risk of bleeding and/or further injury to the kidney. However, it is also unclear how far EVs can travel following a single injection into the parenchyma, since they must diffuse through the extracellular matrix between cells to reach injured target cells. Recently, a modified injection approach through renal subcapsular delivery route has been shown to have better therapeutic effects than the local intraparenchymal injection in an I/R mouse model, providing more widespread resolution of injured renal parenchyma and good renal function recovery.^57,192^ However, once injected (local or subcapsular route) cells or EVs may be washed out into circulation, or into the lymphatic system. Biomaterials such as hydrogels or polymer scaffolds can be used to extend retention of cells, EVs, or other biological therapeutics and provide controlled, prolonged, release at a direct injection site.^193,194^ Lastly, it should be mentioned that a great deal of research explores human cell-derived EVs in mouse models of AKI; thus, immunogenicity cannot be ruled out, especially with repeated systemic administrations of EVs.

### EV Dose, Timing, and Combination Therapies

With regards to dose, the effective amount of EVs per injection for treatment in humans is difficult to estimate. As described earlier, dosing based on particle number or total EV protein concentration is problematic, since these depend on EV purity. The concentrations of purported active substances (key miRNAs, proteins, etc.) are also rarely known or quantified. The weight of an adult human kidney (∼150 g) is ∼200 times greater than an adult mouse kidney, and doses described in preclinical literature vary greatly, as illustrated in Table 1. In theory, the required amount of EVs can be significantly reduced if delivery efficiency can be improved, such as by intra-renal arterial administration, or if cell-type-specific targeting delivery with engineered EVs is utilized.^57^ The optimal timing of therapy following injury is also an important research question. A meta-analysis of studies using animal models found that administration of MSCs >24 h after injury produced improved kidney function (as measured by lower serum creatinine) compared to early (<24 h) administration. Intra-arterial injection also resulted in better outcomes than intravenous or direct intrarenal injection.^195^ This may be due to differences in uptake and retention of MSCs by the injury site, which are affected by the injury and administration route. Since MSC therapeutic effects are driven by paracrine secretions, it is likely that MSC-EVs would share the same optimal therapeutic timing.

### Outlook for EV-based Therapies of AKI

For AKI therapeutic applications, EVs should be considered a combination therapy, delivering a complex cocktail of multiple active ingredients to act on many pathways in different recipient cell types. This is clearly illustrated in Table 1 and Figure 1. However, there are several fundamental factors that are currently limiting their clinical translation. First, there is considerable uncertainty regarding the “optimal” source of EVs (MSCs, non-MSCs, serum, plasma, platelets, etc.), and there are methodological limitations with the methods used to extract and characterize them. As illustrated by examples given earlier, published research has reported quite different properties for EVs in terms of physical parameters, protein expression, and miRNA cargo. There is also a lack of standardization in isolation methods, injury models, doses, and other key variables across studies. This makes it challenging to compare results and determine optimal sources and doses of EVs.

Studies have attributed therapeutic effects of EVs to many different constituents of their cargo (mRNA, multiple miRNAs, surface proteins, etc.) and many different downstream pathways. Thus, there are no current biomarkers for determining whether a given EV population would be beneficial, ineffective, or detrimental in AKI. This illustrates why understanding the functions of individual miRNAs and their combinatorial effects, is important, since it would allow their presence/absence to be used as a screening tool. Again, systems biology-based multi-omic approaches can be useful in this regard to establish desired parameters, such as minimum required concentrations of key components. Related to this, many studies showed equivalent outcomes when delivering miRNAs and whole EVs, in which case providing the miRNA alone would be a much simpler and more efficient therapeutic approach. This is especially relevant if the “active ingredient” miRNA constitutes only a small portion of total EV miRNA, or is perhaps contained within only a small percentage of the total EVs.^100,105^ In addition, if essential miRNA targets and intracellular pathways can be identified with accuracy, siRNAs could be developed to achieve the same results. The US FDA, UK MHRA, and other regulatory bodies have approved siRNA-based drugs for several indications, demonstrating their feasibility as therapeutics. Thus, in our opinion, MSC-EVs from cultured primary cells are overall unlikely to serve as a gold standard treatment for AKI. However, research in this area is still essential to increase our understanding of the underlying biology, which can aid in identifying new therapeutic modalities.

## Conclusion

Cell-derived EVs clearly have great potential in AKI therapy and their activity has been consistently demonstrated in multiple animal models. Given their natural role as dynamic mediators of paracrine effects and cargo of biologically active substances, there is also strong biological plausibility for their therapeutic use. However, the field is still immature in many aspects. There is insufficient biological understanding of EV biological effects, particularly in terms of which cargo components are essential for their activity, or which may act in combination with one another. Multi-omic approaches can assist with this to provide a better understanding of EV cargo and bioactivity and to determine what constitutes an optimal EV for AKI therapy. There is currently a lack of standardization or agreed-upon best practices, and most research articles use varying EV isolation methods, doses, timings, and administration routes, many of which are not easily translated to human patients. Moving forward, we hope to see greater consideration of clinical realities and more translationally relevant models in therapeutic research of AKI.

## Ethical Approval

This review article does not contain any data or information that need specific ethical approval, permissions, or licenses.

## Data Availability

Data is available on request from the authors.
